# Genesis of Preeclampsia: An Epidemiological Approach

**DOI:** 10.5402/2012/916914

**Published:** 2012-02-08

**Authors:** Jaime Salvador-Moysén, Yolanda Martínez-López, José M. Ramírez-Aranda, Marisela Aguilar-Durán, Alberto Terrones-González

**Affiliations:** ^1^Scientific Research Institute, Universidad Juárez del Estado de Durango, Avenida Universidad y Volantín no Number, 34000 Durango, DGO, Mexico; ^2^Department of Family Medicine, University Hospital Dr. José Eleuterio González, Universidad Autónoma de Nuevo León, 64460 Monterrey, NL, Mexico

## Abstract

There are analyzed some of the main aspects related to the causality of preeclampsia, privileging two types of models: the clinic model and the epidemiologic model, first one represented by the hypothesis of the reduced placental perfusion and the second one considering the epidemiologic findings related to the high levels of psychosocial stress and its association with preeclampsia. It is reasoned out the relevance of raising the causality of the disease from an interdisciplinary perspective, integrating the valuable information generated from both types, clinical and epidemiologic, and finally a tentative explanatory model of preeclampsia is proposed, the subclinical and sociocultural aspects that predispose and trigger the disease are emphasized making aspects to stand out: the importance of reduced placental perfusion as an indicator of individual risk, and the high levels of physiological stress, as a result of the unfavorable conditions of the psychosocial surroundings (indicator of population risk) of the pregnant women.

## 1. Background

The several research studies that have been made—practically all over the world—to clarify the causality of preeclampsia have not reached their goal, although they have yielded a more precise knowledge about the mechanisms related to the pathophysiological processes of disease as well as the identification of different risk factors and the emergence of new therapeutic measures [1–11]. The absence of a universally accepted explicative model about the preeclampsia genesis is due to various reasons, among them, it is important to mention the complexity of the disease, universal distribution with variable risk factors at different latitudes in different ethnic groups, and diversity-documented risk indicators that vary depending on whether the approach is epidemiological, clinical, or basic. Usually, the research related with clinical-pathological processes is performed by examining and scrutinizing features related with individual causal factors, in this respect, the modern molecular research techniques have allowed a scrutiny on biological processes that would have been unimaginable some years ago. The research on preeclampsia is within the same logic, which has allowed the clarification of diverse molecular processes related to the disease appearance, even when the disease causality remains enigmatic. It is important to reestablish the necessity to, simultaneously to the meticulous study of the biochemical and molecular processes related to the pathophysiology mechanism of the disease, investigate in detail the population features—of pregnant women groups—of socioepidemiological and psychosocial nature, that represent the vulnerability framework or sociocultural protection of pregnant women. This framework is potentially the trigger element for the expression of individual risk factors—genetic, constitutional, and obstetric—leading to clinical manifestation of preeclampsia. The statement that the sociocultural framework of the gestational women could represent the triggering element for the preeclampsia expression, which is based equally on empirical studies [12–14]—that show the relation of unfavorable psychosocial conditions with a higher disease occurrence—as on theoretical approaches that causally link the stress perception originated by the daily human interaction situations with characteristic neurochemical alterations of physiological stress [15–20]. In this respect, it is convenient to consider the pertinence of the conceptual and methodological approaches that have been made to elucidate the preeclampsia genesis. The theoretical scientists recognize that the classic empirical studies, characterized by an unidisciplinary approach, have not been the best strategy for the complex problem solutions. From their point of view, the most desirable is to make interdisciplinary studies that allow a wider exploration from different perspectives, with a greater possibility of establish clear causal relationships with a better explicative capacity [21]. In reference to preeclampsia, the different perspectives are represented primarily by the results obtained with different methodological procedures—that implicate different conceptual approaches—in such a manner that the obtained information through diverse clinical, basic, and epidemiological study designs represents a valuable, though fragmented, empirical, and conceptual wealth that would be worthy to articulate with organizational cognitive purposes, in order to search for possible relationships between the diverse findings, that allow a wider and more coherent vision of the disease and of the multidimensional features that precede and characterize it.

From the various methodological approaches—of epidemiological nature—to identify the causal sequence of preeclampsia, we must cite the one concerning investigations related to the perception of the psychosocial environment that the pregnant woman has and its impact on health conditions through the gestational process and specifically on the association with the toxemia expression. There are numerous studies that clearly document the association between the psychosocial stressors presence, the lack of psychosocial support, and a higher frequency of preeclampsia within diverse work, social, and hospital environments [22–28]. The association of anxiety conditions—as a consequence of individual psychological characteristics—with the disease appearance has also been documented [29, 30]. The evidence showed by Ringrose in 1961 [31] using the Minnesota Multiphasic Personality Inventory (MMPI) implicates personality decompensation during pregnancy resulting from stress as the etiological mechanism in toxemia of pregnancy. The author's clarity about the importance of the emotional in the occurrence of the disease is the most significant of his study, even when the instrument used has not been the most appropriate one, an understandable situation regarding that is a study from 50 years ago and that the psychosocial stress measurement constructs that we nowadays have, and that have been designed to be applied to a healthy population, did not exist. The individual psychosocial aspects jointly with the sociocultural context of gestational women represent an inquiry ground that has to be studied in depth. It is important to highlight the identification of a population common denominator—psychosocial stress—that can be produced by various circumstances; economic adversity, work difficulties, family or social pressures, affective or emotional conflicts; and that when coexists with a lack of psychosocial support has showed a consistent association with the preeclampsia expression. Consequence of this findings has been the elaboration and validation of constructs [32, 33] in order to evaluate in a reliable manner the presence and magnitude of both, psychosocial stressors—stressors are represented by those familiar, work, economic, social, and cultural situations that require and exceed the personal resources of the individual in such a manner that represent threat, harm, or challenge—and psychosocial support—which is represented by the social and familiar environment from which the individual satisfies its needs for social, affective, communication, solidarity, and economic acknowledgement. It is important to mention that there are practically no empirical studies that connect or integrate these relevant epidemiological findings to the clinical research designs directed to solve the preeclampsia causality question. The omission or underutilization of epidemiological indicators in clinical or basic approaches—even when there is no explication to do it—could obey to that they seem to be far from clinical manifestations and molecular processes of the disease, even though there is also another perspective that lets place population indicators as nonspecific predisposing factors that precede temporarily individual risk factors, which are closely linked to them and are able to determine the clinical expression of disease. A plausible explanation for this arguments is found in the works on allostasis—physiological processes that allow the organism to achieve stability through change—and allostatic load developed by Bruce McEwen. He argues that the failed response to chronic stress can promote and exacerbate pathophysiological processes through the derailment of immunological, cardiovascular, metabolic, and neural mechanisms, which are often accompanied by changes in personal behavior, such as poor diet, lack of exercise, and lack sleep. The allostatic load is represented by the coexistence of inefficient homeostatic mechanisms and inappropriate personal behavior of lifestyle; it is the result of the sum of unfavorable sociocultural situations that the individual is unable to cope successfully, and that predisposes the expression of pathology due to the diminished capacity of biological resistance resulting from alterations of physiological adaptive mechanisms [19, 20]. These approaches that are rooted in the classic pioneering work of Selye [15, 16] and have been developed by several authors, among which Milsum [34] and Cassel [35] must be cited that have reached a high level of scientific clarity and strength in McEwen's contributions, which can be considered as a powerful conceptual tool for addressing a variety of health problems unsolved, among which preeclampsia must be included. 

## 2. Problem Location

From an epidemiological approach, causal models consider different types of risk factors according to their temporal location in relation with the occurrence of the disease; from this view, we can mention, among others, the predisposing factors and the precipitating ones [36]. The former may precede by weeks, months, or even years the onset of the disease; precipitating factors, instead, are the immediate predecessor of the clinical expression of a pathological process. The application of this simple epidemiological scheme to investigation studies on the genesis of preeclampsia, let us see in a more complete and comprehensive way the diversity of variables and its attempt interaction at different times of the natural history of disease, from which it is essential to explore more widely the subclinical stage, since the identification of early risk indicators would enable the timely implementation of interventions to minimize the severity of the disease, and even their prevention. The existence of rigorous results obtained on preeclampsia, both clinical and epidemiological, does not guarantee the appropriate conceptual or methodological articulation of the findings obtained with both approaches, since the nature of the concerning individual or population is different and consequently their conceptual frames and methodological strategies also differ [37]. It is important to seek any theoretical or conceptual element that would be able to articulate coherently the epidemiological findings with clinical evidence, so that the diversity of clinical and epidemiological data form an articulated and coherent corpus capable of appreciating more clearly the sequence of events that occur in preeclampsia, in both subclinical and clinically manifested stage, that is, the natural history of this disease would have to be reconfigured, strengthening the cognitive aspects that precede its clinical expression.

## 3. Explanatory Models of Preeclampsia

### 3.1. Clinical Model

A two-stage model of preeclampsia has been proposed, as a useful conceptual tool to explain its pathophysiology. In the first stage and as the source of the problem, the failure of placentation has been pointed out. Among the different molecules involved in placental neovascularization; vascular endothelial growth factor receptors (VEGFRs) and type I receptor of angiotensin II (AT1) mediate important molecular signaling pathways of the maternal circulatory system and fetal growth. VEGFR1 and VEGFR2 are functional receptors for the placental growth factor (PLGF) and vascular endothelial growth factor, respectively; when the interactions established between these molecules are altered, the molecular signaling pathways aimed to maintain homeostasis are also modified. It has been suggested that altering the balance between angiogenic and antiangiogenic substances signalization is closely related to the occurrence of preeclampsia [11, 38]. From this condition, some women pass to the second stage: systemic dysfunction, in which is exacerbated the hypoxia of the placenta and typical signs of this clinical condition, such as hypertension, proteinuria, coagulation problems, and liver dysfunction, are expressed. It should also be mentioned that, in obstetric conditions characterized by excessive placental tissue, such as molar pregnancy and multiple gestation, the frequency of the disease is increased. Importantly, reduced placental perfusion is not unequivocally associated with the disease expression, as there is a variable proportion of women who have no clinical symptoms, therefore, it is feasible to consider that reduced placental perfusion *per se* is not a determinant of disease, except in extreme situations, because they must interact with other maternal characteristics for preeclampsia to be clinically expressed.

Investigations oriented to go deep on individual variables of pregnant women—genetic, constitutional, obstetric—tentatively associated with the genesis of preeclampsia have contributed original and valuable information about the different pathophysiological mechanisms of disease, although its contribution has not been significant to clarify the causal network.

### 3.2. Epidemiological Model

Of the various approaches made to the epidemiological problem of the genesis of preeclampsia, it is important to highlight the one related to the valuation of psychosocial environment of pregnant women, considering that pregnancy demands adaptive efforts not only physical but also emotional and psychosocial. Different studies have documented the association of adverse psychosocial and cultural conditions, with a higher frequency of morbidity and mortality associated to gestational process [39, 40]. Empirical approaches establishing a clear association between a sociocultural or institutional context, characterized by high levels of psychosocial stressors and low psychosocial support, with the clinical expression of preeclampsia [22, 24–26, 28]. These unfavorable psychosocial conditions can be considered, from an epidemiological perspective, as predisposing as well as precipitating factors of the disease, which depends on the pregnant women's timing of exposure to psychosocial stress conditions. It is logical to suggest that the unfavorable sociocultural context of pregnant women is a “handicap.” This is expressed clinically as an “disability” anatomically located in the hypothalamic-pituitary-adrenal (HPA) axis leading to a chronic physiological stress response that favors the expression of preeclampsia in women with individual risk factors—genetic, constitutional, and obstetric—primarily due to the derailment of the homeostatic mechanisms. In reviewing the information related to the disadvantages of the sociocultural context and its association with preeclampsia, we see that the observations are located during development of the gestational process, from the early stages of pregnancy, almost to the expression of the disease. However, according to data from different studies, it can be inferred that unfavorable psychosocial stress conditions or sociocultural environment precede pregnancy. Epidemiological information is of a significant importance, mainly in the subclinical period of preeclampsia, due to the identification of tentative population risk factors shown to be consistent in different empirical studies. Although the detailed analysis of each of the indicators, both clinical and epidemiological, associated with the genesis of preeclampsia is beyond the scope of this document, you may refer some of the most important ones according to what is documented in various studies and shown schematically through a tentative causal model of preeclampsia.

## 4. Proposal of an Explanatory Model of Preeclampsia

**Figure 1:**
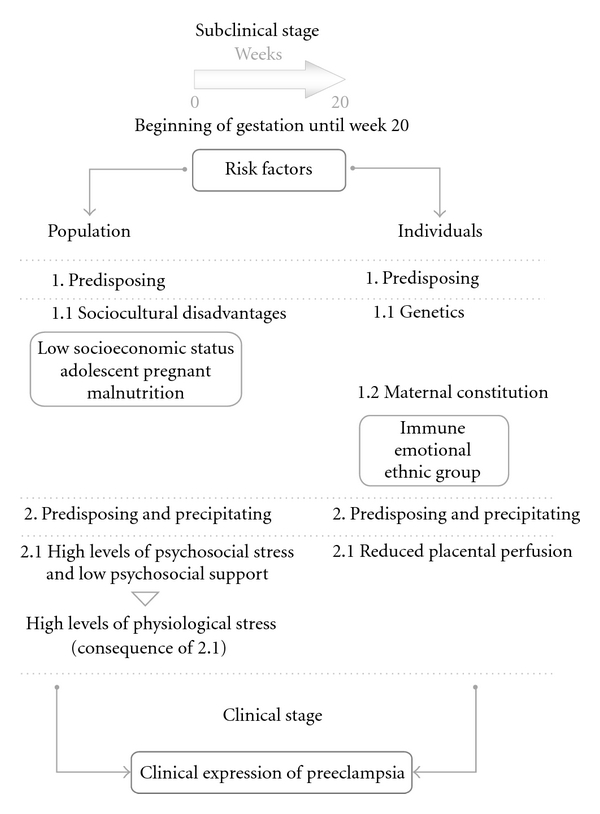


The proposal schematized in Figure 1 has been built upon some elements of the Natural History of Disease. The aspects highlighted are located in the subclinical stage as they represent the potential risk factors associated with the expression of preeclampsia and tentatively are part of its causal network. The columns of the risk factors include well-documented aspects of both population-related and clinical in nature; it is noteworthy that both factors are present not only at the beginning of pregnancy but even before it. With regard to the triggers of the disease, both reduced placental perfusion and high levels of physiological stress postulated as important conditions for the clinical expression of disease. We believe that the development of studies weighing up and defining the interaction of population with clinical risk factors will yield clearer and more accurate information about the genesis of preeclampsia. Evaluating the epidemiological variables and their role as predisposing factors and triggers of the disease, there are results we have obtained in various empirical studies in different cities of Mexico with groups of pregnant adolescents, in which the risk of falling ill with preeclampsia is observed in groups of pregnant adolescents with low socioeconomic status, who showed high levels of psychosocial stress and low psychosocial support was 2.5 to 4 times higher, than in the group of adolescents with the same socioeconomic level, but with significant psychosocial support [24, 26, 41]. We also noted that with values >13.9 nmol/L of salivary cortisol obtained before 20 weeks of gestation, the risk of falling ill was 1 (100%) and with values ≤13.9 nmol/L of salivary cortisol, the probability of not getting ill was 1 (100%) [42]. 

The tentative preeclampsia causal model presented was constructed with empirical data obtained by our working group (socioepidemiological aspects and salivary cortisol) with data reported in the international literature (epidemiological and clinical) and with Bruce McEwen's conceptual contributions as theoretical support. We consider the model applicable to groups of pregnant teens not only in Mexico but in countries with similar sociocultural characteristics in which teenage pregnancy is common and represents a strong social and emotional pressure. The epidemiological aspects already underpinned in studies documented in various publications are the ones related to psychosociocultural environmental partnership with the expression of preeclampsia. A constant in these works strongly suggests that although both the geographical context and the economic and educational status of pregnant women differ, perceived chronic stress represents a common element that precedes the expression of the disease and should be regarded with greater attention in both clinical and epidemiological studies aimed at establishing causation of preeclampsia. The least explored aspect to be empirically validated is the usefulness of cortisol as a predictor of preeclampsia. We have preliminary results from an empirical approach carried out in a cohort of 100 pregnant adolescents (measuring salivary cortisol) in which we obtained negative and predictive values of 1 (100%) for both estimates [42]. 

Although the results we obtained with salivary cortisol, as a predictor of preeclampsia in pregnant adolescents, are contradictory with the data obtained by other authors in elderly women [43, 44], does not mean that they lack validity. There are several empirical evidences and quasi-experimental studies that show a clear association between stress conditions and salivary cortisol high values [45–47]. A tentative explanation of these inconsistencies can be sustained on clinical and age differences that exist between adolescents and elderly women group, which may determine a different neurophysiological response. In stressful situations, healthy pregnant adolescents, are expected to have an effective adaptative response that is accompanied by increased cortisol levels; if the stress is a cronical condition, it prevents the cortisol returns to normal values and becomes a risk factor [19]. In the group of elderly women with preeclampsia it happens different, as is frequent that they present pathological processes that are associated with preeclampsia, and often preeclampsia is accompanied by complications, conditions that represent biological stress that by themselves could determine an inefficient neurophysiological response with low values of cortisol.

## 5. Conclusion

The numerous studies conducted to understand the causal network of preeclampsia, its diversity of approaches, and variety of indicators identified in some way associated with the disease have made extraordinarily difficult the task of systematizing the information and proposing an explanatory model with universal acceptance. The simple model here proposed is not intended to fully explain the series of events that occur in pregnant women prior to clinical manifestation of the disease but aims to propose a tentative explanation of the causal sequence—mainly in groups of adolescent mothers—rigorously supported by information obtained, which privileges the risk factors of psychosociocultural nature, prevailing both in early pregnancy and in pregestational stage, which are associated with socioeconomic disadvantage conditions (although not necessarily) and that are closely linked to psychophysiological stress conditions, which encourage the expression of individual risk factors and clinical manifestation of preeclampsia.

## References

[1] López-Llera M (1995). Complexity and complicity in eclampsia: barriers or bridges?. *Medical Hypotheses*.

[2] Dekker G, Sibai B (2001). Primary, secondary, and tertiary prevention of pre-eclampsia. *The Lancet*.

[3] Roberts JM, Cooper DW (2001). Pathogenesis and genetics of pre-eclampsia. *The Lancet*.

[4] Luft FC (2003). Pieces of the preeclampsia puzzle. *Nephrology Dialysis Transplantation*.

[5] Podjarny E, Losonczy G, Baylis C (2004). Animal models of preeclampsia. *Seminars in Nephrology*.

[6] Patrick TE, Powers RW, Daftary AR, Ness RB, Roberts JM (2004). Homocysteine and folic acid are inversely related in black women with preeclampsia. *Hypertension*.

[7] Redman CW, Sargent IL (2005). Latest advances in understanding preeclampsia. *Science*.

[8] Rudra CB, Williams MA (2005). Monthly variation in preeclampsia prevalence: Washington State, 1987–2001. *Journal of Maternal-Fetal and Neonatal Medicine*.

[9] Sibai B, Dekker G, Kupferminc M (2005). Pre-eclampsia. *The Lancet*.

[10] Myatt L, Webster RP (2009). Is vascular biology in preeclampsia better?. *Journal of Thrombosis and Haemostasis*.

[11] Grill S, Rusterholz C, Zanetti-Dällenbach R (2009). Potential markers of preeclampsia-a review. *Reproductive Biology and Endocrinology*.

[12] Sheehan TJ (1998). Stress and low birth weight: a structural modeling approach using real life stressors. *Social Science and Medicine*.

[13] Butler LD, Koopman C, Classen C, Spiegel D (1999). Traumatic stress, life events, and emotional support in women with metastatic breast cancer: cancer-related traumatic stress symptoms associated with past and current stressors. *Health Psychology*.

[14] Wamala SP, Mittleman MA, Horsten M, Schenck-Gustafsson K, Orth-Gomér K (2000). Job stress and the occupational gradient in coronary heart disease risk in women: the Stockholm Female Coronary Risk Study. *Social Science and Medicine*.

[15] Selye H (1955). Stress and disease. *Science*.

[16] Selye H (1970). The evolution of the stress concept. Stress and cardiovascular disease. *The American Journal of Cardiology*.

[17] Cohen S, Wills TA (1985). Stress, social support, and the buffering hypothesis. *Psychological Bulletin*.

[18] McEwen BS (2005). Stressed or stressed out: what is the difference?. *Journal of Psychiatry and Neuroscience*.

[19] McEwen BS, Mirsky AE, Hatch H, Hatch M (2007). Physiology and neurobiology of stress and adaptation: central role of the brain. *Physiological Reviews*.

[20] McEwen BS (2008). Central effects of stress hormones in health and disease: understanding the protective and damaging effects of stress and stress mediators. *European Journal of Pharmacology*.

[21] Bunge M (2003). *Emergencia y Convergencia: Novedad Cualitativa y Unidad del Conocimiento*.

[22] Klebanoff MA, Shiono PH, Rhoads GG (1990). Outcomes of pregnancy in a national sample of resident physicians. *The New England Journal of Medicine*.

[23] Landsbergis PA, Hatch MC (1996). Psychosocial work stress and pregnancy-induced hypertension. *Epidemiology*.

[24] Salvador-Moysén J, Martínez-López Y, Lechuga-Quiñones A, Ruiz-Astorga R, Terrones-González A (2000). Psychosocial conditions of adolescents with toxemia of pregnancy. *Salud Publica de Mexico*.

[25] Cerón-Mireles P, Harlow SD, Sánchez-Carrillo CI, Núñez RM (2001). Risk factors for pre-eclampsia/eclampsia among working women in Mexico City. *Paediatric and Perinatal Epidemiology*.

[26] Salvador J, Martínez Y, Lechuga A, Terrones A (2005). Hipertensión inducida por el embarazo en adolescentes: un estudio multicéntrico. *Ansiedad y Estrés*.

[27] Herrera JA, Gao E, Shahabuddin AKM (2006). Periodical assessment of the prenatal biopsychosocial risk to predict obstetric and perinatal complications in Asian countries 2002-2003. *Colombia Medica*.

[28] Abeysena C, Jayawardana P, Seneviratne R (2010). Effect of psychosocial stress on maternal complications during pregnancy: a cohort study. *International Journal of Collaborative Research on Internal Medicine & Public Health*.

[29] Teixeira JMA, Fisk NM, Glover V (1999). Association between maternal anxiety in pregnancy and increased uterine artery resistance index: Cohort based study. *British Medical Journal*.

[30] Terrones A, Salvador J, Lechuga AM, Martínez Y, Garvalena MJ, Nápoles C (2003). Diferencias en ansiedad estado-rasgo entre adolescentes con hipertensión inducida por el embarazo y adolescentes embarazadas sanas. *Ansiedad y Estrés*.

[31] Ringrose C (1961). Psychosomatic influences in the genesis of toxemia of pregnancy. *Canadian Medical Association Journal*.

[32] Cervantes R, Castro F (1985). Stress, coping and Mexican-American mental health: a systematic review. *Hispanic Journal of Behavioral Sciences*.

[33] Furukawa T, Goldberg DP (1999). Cultural invariance of likelihood ratios for the General Health Questionnaire. *The Lancet*.

[34] Milsum JH (1985). A model of the eustress system for health/illness. *Behavioral Science*.

[35] Cassel J (1974). Psychosocial processes and ’stress’: theoretical formulation. *International Journal of Health Services*.

[36] Last JM (2001). *A Dictionary of Epidemiology*.

[37] Susser M (1991). *Conceptos y Estrategias en Epidemiología. El Pensamiento Causal en las Ciencias de la Salud*.

[38] Furuya M, Kurasawa K, Nagahama K (2011). Disrupted balance of angiogenic and antiangiogenic signaling in preeclampsia. *Jounal of Pregnancy*.

[39] Nuckolls KB, Cassel J, Kaplan BH (1972). Psychosocial assets, life crisis and the prognosis of pregnancy. *American Journal of Epidemiology*.

[40] Erickson MT (1976). The relationship between psychological variables and specific complications of pregnancy, labor, and delivery. *Journal of Psychosomatic Research*.

[41] Salvador Moysén J (2009). The biopsychosocial dimension of preeclampsia: a conceptual-empirical approach. *Ginecologia y Obstetricia de Mexico*.

[42] Salvador-Moysén J, Ramírez-Aranda JM, Martínez-López Y, Aguilar Durán M (2012). Salivary cortisol levels as a predictor of preeclampsia in adolescents. *Colombia Médica*.

[43] Sikkema JM, Robles de Medina PG, Schaad RR (2001). Salivary cortisol levels and anxiety are not increased in women destined to develop preeclampsia. *Journal of Psychosomatic Research*.

[44] Ho JT, Lewis JG, O’Loughlin P (2007). Reduced maternal corticosteroid-binding globulin and cortisol levels in pre-eclampsia and gamete recipient pregnancies. *Clinical Endocrinology*.

[45] De Weerth C, Graat G, Buitelaar JK, Thijssen JHH (2003). Measurement of cortisol in small quantities of saliva. *Clinical Chemistry*.

[46] De Weerth C, Wied CC, Jansen LM, Buitelaar JK (2007). Cardiovascular and cortisol responses to a psychological stressor during pregnancy. *Acta Obstetricia et Gynecologica Scandinavica*.

[47] Covelli MM (2007). Prevalence of behavioral and physiological risk factors of hypertension in African American adolescents. *Pediatric Nursing*.

